# Polymerase chain reaction of fistula drainage in sacrococcygeal tuberculosis

**DOI:** 10.1016/j.ijscr.2019.07.057

**Published:** 2019-07-30

**Authors:** Patrick M. Foye, Manpreet Bains, Vidur Tangri

**Affiliations:** Rutgers New Jersey Medical School 90 Bergen St, D.O.C. Suite 3100, Newark, NJ, 07103-2425, United States

We praise your journal and authors Takakura, et al., on the excellent article titled, “Rare case of sacrococcygeal tuberculosis mimicking as an anal fistula” [1].

While Pott disease (also known as tuberculous spondylitis) usually involves the thoracolumbar spine, Takakura, et al., present a fantastic example of the rare involvement of the sacrum and coccyx. Their diligent and persistent diagnostic workup was very impressive. Specifically, the patient had no pulmonary symptoms, a normal chest radiograph, and cultures from the fistula discharge were negative for bacteria and also negative for acid-fast bacilli (AFB). Yet the clinicians persisted in doing additional testing of the drainage via polymerase chain reaction (PCR), which was positive for Mycobacterium tuberculosis, thus confirming sacrococcygeal TB. Treating the TB resolved the patient’s pain, discharge, and fluid collection, thus providing an excellent outcome.

The authors also provided an excellent literature review. We humbly suggest adding one additional citation on this topic. Specifically, Lougheed and White in 1960 reported performing coccygectomy to drain purulent TB fluid located anterior to the sacrum/coccyx, which had arisen from gravity-dependent pooling from TB involving the L5-S1 vertebral levels [2]. Takakura, et al., apparently did not need to resort to such surgical removal of the coccyx, perhaps due to better multi-drug anti-tuberculosis antibiotic protocols than were available in 1960.

## Declaration of Competing Interest

None.

## Funding

None.

## Ethical approval

N/A.

## Consent

N/A.

## Author contribution

Because this is a letter to the editor, there is no data collection, data analysis/interpretation. The authors of the letter each contributed to the concept/design/writing/reviewing of the letter.

## Registration of research studies

N/A.

## Guarantor

Patrick M. Foye, M.D.
